# Cytokine levels as predictors of mortality in critically ill patients with severe COVID-19 pneumonia: Case-control study nested within a cohort in Colombia

**DOI:** 10.3389/fmed.2022.1005636

**Published:** 2022-09-29

**Authors:** Francisco José Molina, Luz Elena Botero, Juan Pablo Isaza, Luz Elena Cano, Lucelly López, Lina Marcela Hoyos, Elizabeth Correa, Antoni Torres

**Affiliations:** ^1^Facultad de Medicina, Escuela de Ciencias de la Salud, Universidad Pontificia Bolivariana, Medellín, Colombia; ^2^Intensive Care Unit, Clínica Universitaria Bolivariana, Universidad Pontificia Bolivariana, Medellín, Colombia; ^3^Corporación para Investigaciones Biológicas, Medellín, Colombia; ^4^Department of Pulmonology, University of Barcelona, Barcelona, Spain; ^5^Respiratory and Intensive Care Unit, Hospital Clinic of Barcelona, Barcelona, Spain

**Keywords:** COVID-19, SARS-CoV-2, cytokines, mortality, intensive care units, pneumonia

## Abstract

**Background:**

High levels of different cytokines have been associated in COVID-19 as predictors of mortality; however, not all studies have found this association and its role to cause multi-organ failure and death has not been fully defined. This study aimed to investigate the association of the levels of 10 cytokines with mortality in patients with COVID-19 admitted to the intensive care unit (ICU).

**Materials and methods:**

This is a case-control study nested within a cohort of patients with COVID-19 who were on mechanical ventilation and were not hospitalized for more than 48 h across nine ICUs in Medellín, Colombia. Serum samples were collected upon admission to the ICU and 7 days later and used to measure cytokine levels.

**Results:**

Upon admission, no differences in mortality between the cytokine levels were observed when comparisons were made quantitatively. However, in the multivariate analysis, patients with median IL-1β levels <1.365 pg/ml showed an increase in mortality (OR = 3.1; 1.24<7.71; *p* = 0.015). On day 7 in the ICU, IL-1β median levels were lower (0.34 vs. 2.41 pg/ml, *p* = 0.042) and IL-10 higher (2.08 vs. 1.05 pg/ml, *p* = 0.009) in patients who died. However, in the multivariate analysis, only IL-12p70 was associated with mortality (OR = 0.23; 0.07<0.73; *p* = 0.012). The mean difference in the levels between day 1 and day 7 decreased in both IFN-*γ* (3.939 pg/ml, *p* < 0.039) and in IL-18 (16.312 pg/ml, *p* < 0.014) in the patients who died. A low IL-1β/IL-10 ratio was associated with mortality on both day 1 and day 7, while an IL-1β/IL-10 ratio below the cut-off on day 7 was associated with decreased survival. The lowest TNFα/IL-10 ratio was associated with mortality only on day 7.

**Conclusion:**

At the time of admission, patients with median IL-1β levels lower than 1.365 pg/ml had increased mortality. An IL-1β/IL-10 ratio <2 at day 7 and IL-12p70 levels >1.666 pg/ml was associated with decreased survival.

## Introduction

The coronavirus disease 2019 (COVID-19/SARS-CoV-2) pandemic has resulted in high rates of mortality (43% [95% CI: 0.29<0.58]) in patients with invasive mechanical ventilation (IMV) in intensive care units (ICUs) (1). The global UNITE-COVID study found that older age, IMV, and acute kidney injury (AKI) were the strongest predictors of mortality (2). The “cytokine storm” (CS) is characterized in severe COVID-19 by systemic inflammation with increased ferritin, D-dimers, C-reactive protein (CRP), and cytokines such as tumor necrosis factor-alpha (TNFα), interleukin 1 beta (IL-1β), interleukin-6 (IL-6), interferon-gamma (IFN-*γ*), and interleukin 18 (IL-18), that leads to multi-organ failure (3).

Ruan et al. found that ± 45% (68/150) of patients hospitalized in two centers in Wuhan, China, for infection of SARS-CoV-2, had died, and had higher levels of IL-6 (4). Fernandez-Botran et al. reported that high levels of IL-6 in hospitalized patients with COVID-19 were associated with the need for ICU admission, and the use of vasopressors (5). Milenkovic et al. in a retrospective cohort study of 318 patients admitted to an ICU in Belgrade, Serbia discovered that IL-6 ≥74.98 pg/ml was a predictor of mortality (6). Sancho Ferrando et al. in 122 patients with COVID-19 at ICU admission, found that the levels of TNFα receptors in their soluble form (sTNFR) 1 and 2 were higher in those who developed acute kidney injury (AKI) and in those who did not survive 30 days (7). McElvaney et al. reported that patients admitted to the ICU had higher values of IL-1β, IL-6, IL-8, and IL-10 (8).

However, Kox et al. described that plasma concentrations of tumor necrosis factor (TNF), IL-6, and IL-8 taken in the first 24 h of ICU admission were lower in patients with acute respiratory distress syndrome (ARDS) in IMV with COVID-19 than in patients with septic shock with or without ARDS and comparable to patients with out-of-hospital cardiac arrest and multiple traumas (9). Bülow Anderberg et al. showed a strong correlation at the time of admission to ICU between the lowest PaO2/FiO2 ratio and increased levels of IL-1Ra, IL-4, IL-6, and IL-8 in 24 adults with COVID-19. There was also a strong correlation of several biomarkers such as IL-4, IL-6, IL-8, IL-10 and TNFα with acute kidney injury; however, cytokines were weakly correlated with mortality except for IL-8 (10).

Li et al. in a study conducted in 40 patients hospitalized in an ICU in Wuhan, China, described how the kinetic variations in IL-6, IL-8, and IL-10 levels were associated with mortality (11). Tong-Minh et al. reported that an increase in procalcitonin (PCT), IL-6, and soluble urokinase-type plasminogen activator receptor (suPAR) in a subsequent day of ICU stay were predictors of in-hospital mortality (12). The dynamics of cytokine release during severe COVID-19 remain unknown. Moreover, whether a CS in severe COVID-19 is the most apt description of the pathogenesis remains to be determined.

We therefore focus on the critically ill patient where the levels of separate cytokines may give only a partial view of a complex and interrelated inflammatory process in the patient with COVID-19. We hypothesized that the plasma levels of cytokines are elevated like other inflammatory mediators in severe SARS-CoV-2 infection and could serve as a biomarker of multi-organ failure and death. This study aimed to characterize the association of 10 cytokine levels with mortality in patients with COVID-19 admitted to ICU with respiratory failure in IMV of samples taken on days 1 and 7 of ICU. Moreover, we sought to determine whether differences in the blood concentrations of cytokines on days 1 and 7 were associated with mortality.

## Materials and methods

### Study setting

**FIGURE 1:**
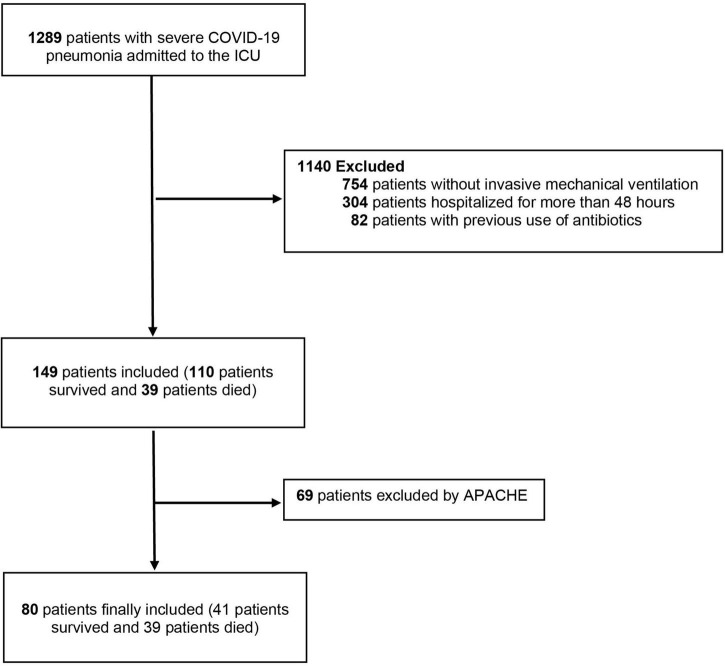
Study flowchart. ICU, intensive care unit; APACHE, Acute Physiology and Chronic Health disease.

This is a case-control study nested within a cohort of 149 patients included in the original study (13). The patients were >18 years of age with confirmed COVID-19 admitted at nine ICUs in Medellín, Colombia. All patients were on mechanical ventilation and could not have been hospitalized for more than 48 h at the time of ICU admission. The study was conducted between March 1 and July 30, 2021. Of the 149 patients, 39 died, and they were matched with 41 surviving patients to obtain an almost 1:1 ratio by Acute Physiology and Chronic Health disease Classification System (APACHE). The total sample size was 80 patients (Figure 1). Clinical data was retrieved from their electronic medical records. Sequential Organ Failure Assessment (SOFA) score (14), and APACHE II (15) data were collected upon ICU admission. Moreover, blood samples were collected on the day 1 of ICU for routine chemistry. Laboratory tests included white blood cell count (WBC), CRP, ferritin, procalcitonin, D-dimer, creatinine, troponin and ferritin.

### COVID-19

The diagnosis of SARS-CoV-2 was confirmed using real-time PCR (RT-PCR) on samples obtained from nasopharyngeal swabs, in an Allplex assay (Seegene, Inc., Seoul, South Korea) to amplify SARS-CoV-2 E, RdRp/s, and N genes.

### Cytokine measurements

Venous blood samples were collected upon admission to the ICU and 7 days later, in order to measure cytokine levels. Cytokines were measured at day 7 to determine their predictive value for mortality after the first week of hospitalization in those who were alive. In addition, to measure the dynamic changes with the same time difference, between days 1 and 7, in the mean difference of the serum levels of each cytokine, and its predictive value with mortality. This could generate hypotheses for possible treatments before the fatal outcome occurs.

Serum was obtained from each blood sample by centrifugation for 10 min at 2,000 RPM. Subsequently, each serum sample was stored in cryovials at –80°C until processing. This process was carried out in the BSL2 Research Laboratory of the Faculty of Medicine of the Universidad Pontificia Bolivariana (Medellín, Colombia). For this investigation, a system Human ProcartaPlex TM Multiplex Immunoassay Mix & Match of 10-Plex based on magnetic beads was selected to detect serum biomarkers Ref. PPX-10-MX323G4 (Invitrogen, Whatman, Massachusetts, United States). These analytes included interleukin 1 beta (IL-1β), IL-2, IL-6, IL-10, IL-12p70, IL-17A, interferon-gamma (IFN-*γ*), recombinant human granulocyte-macrophage colony-stimulating factor (GM-CSF), tumor necrosis factor-alpha (TNFα), and IL-18. Undiluted samples were processed following the manufacturer’ instructions. The 10 cytokines were analyzed using the Luminex^®^ MAGPIX^®^ System (ThermoFisher Scientific, Luminex Corporation 12212 Technology Blvd. Austin, Texas 78727). All samples and standards were measured in duplicate. Primary data were analysed using Xponet ^®^ Software (Luminex, Austin, Texas, United States). This process was done in the Medical and Experimental Micology Laboratory of the Corporación para Investigaciones Biológicas (Medellín, Colombia).

### Ethical approval

This study was approved by the ethics committee of the Universidad Pontificia Bolivariana and by the committees of the Clinics and Hospitals that participated in the study. Written informed consent was obtained from the participants or their legal representatives.

### Statistical analysis

Continuous variables were presented as medians and 25th and 75th percentiles or mean and standard deviation (SD) depending on the distribution of the variables. Categorical variables were presented as frequencies and percentages. In the comparison of cytokines with mortality and other dichotomous variables, the Mann–Whitney *U* test was used. To compare the laboratory parameters, APACHE II, and SOFA, Spearman’s correlation coefficient was used. The change in the difference of serum levels of cytokines between days 1 and 7 with mortality was estimated. We calculated crude and adjusted odds ratios (OR)s with their confidence intervals (CI). Multivariate analysis was performed in a logistic regression model with the cytokines after being dichotomized, taking as cut-off points the medians with a statistical significance of <0.25. To evaluate the pro-inflammatory vs. anti-inflammatory activity, the following ratios were calculated: TNFα/IL-10, and IL-1β/IL-10 (16). Data were analyzed using SPSS version 26.0 and visualized using the ggplot2 and reshape2 libraries.

## Results

### Patient characteristics and cytokine concentrations

The demographic characteristics of the study patients are shown in Table 1. There was higher mortality in men, older age and with a history of hypertension. Table 2 lists the cytokine levels of the 80 patients on the first day of admission, and on day 7 of the 75 patients who were alive. The GM-CSF cytokine did not show any changes after 7 days and was therefore excluded from subsequent analyses.

**TABLE 1:** Demographic characteristics of patients with COVID-19 pneumonia admitted to intensive care units.

Demographic characteristics	Died	Survived	*P*-value
Age–year, m ± sd	62.3 ± 11.6	52 ± 13.5	0.001
Male–no (%)	25 (64.1)	14 (34.1)	0.007
Body mass index, Me–IQR	29.9 (26.9–32.0)	28.4 (24.4–33.1)	0.4
Hypertension–no (%)	22 (56.4)	12 (29.3)	0.014
Diabetes–no (%)	10 (25.6)	9 (22)	0.698
Chronic kidney disease–no (%)	4 (10.3)	1 (2.4)	0.149
Rheumatologic disease–no (%)	1 (2.6)	1 (2.4)	0.971
Neoplasm–no (%)	1 (2.6)	0	0.302
COPD–no (%)	2 (5.1)	0	0.142
HIV–no (%)	0	2 (4.9)	0.162
Chronic liver disease–no (%)	0	1 (2.4)	0.326
COVID vaccination–no (%)	4 (11.1)	2 (5.7)	0.414
Prior steroid use–no (%)	2 (5.6)	1 (3)	0.607
Days of symptoms, Me–IQR	10 (7.0–13.0)	8 (7.0–11.0)	0.610

m, average; sd, standard deviation; no, absolute frequency; %, percentage; Me, median; IQR, interquartile range; COPD, chronic obstructive pulmonary disease; HIV, human immunodeficiency virus.

**TABLE 2:** Levels of the 10 cytokines and association with mortality on days 1 and 7 of ICU stay.

Cytokines	ICU stay	Me (IQR)	Min–Max	Mortality			
				OR crude	*P*-value	OR adjusted	*P*-value
IL-1β	Day 1	1.36 (0.22–2.41)	0.16–2.41	3.10 (1.24–7.71)	0.015	2.79 (1.08–7.22)	0.035
	Day 7	2.41 (0.22–2.41)	0.16–2.41	3.14 (1.22–8.08)	0.017	1.81 (0.60–5.52)	0.295
IL-2	Day 1	4.52 (3.58–5.0)	2.70–5.32	1.00 (0.42–2.41)	0.996		
	Day 7	4.52 (3.19–5.0)	2.44–7.9	1.06 (0.43–2.62)	0.902		
IL-6	Day 1	11.76 (9.63–18.21)	0.74–224.38	0.67 (0.27–1.66)	0.387		
	Day 7	11.76 (11.76–13.58)	2.28–266.62	0.77 (0.27–2.25)	0.638		
IL-10	Day 1	3.21 (1.38–8.92)	0.30–71.24	0.66 (0.27–1.62)	0.366		
	Day 7	1.49 (0.82–6.93)	0.04–260.63	0.41 (0.16–1.05)	0.064	0.47 (0.15–1.48)	0.197
IL-12p70	Day 1	1.71 (1.58–1.98)	1.11–2.12	0.91 (0.37–2.18)	0.823		
	Day 7	1.66 (1.5–1.98)	1.11–2.69	0.36 (0.14–0.93)	0.034	0.23 (0.07–0.73)	**0.012**
IL-17A	Day 1	2.80 (2.80–2.80)	0.26–5.59				
	Day 7	2.80 (2.80–2.80)	1.55–2.80				
IFN-*γ*	Day 1	6.74 (4.58–10.7)	1.26–2.80	2.50 (1.02–3.00)	0.046	1.88 (0.28–12.80)	0.519
	Day 7	4.87 (3.70–6.27)	1.10–18.94	1.93 (0.77–4.88)	0.164	2.08 (0.68–6.30)	0.198
GM-CSF	Day 1	18.67 (18.67–18.67)	18.67–18.67				
	Day 7	18.67 (18.67–18.67)	18.26–18.67				
TNF-α	Day 1	3.77 (1.93–8.64)	0.44–14.05	1.35 (0.56–3.25)	0.503		
	Day 7	4.54 (1.80–8.64)	0.44–27.03	1.76 (0.69–4.48)	0.239	2.16 (0.74–6.13)	0.161
IL-18	Day 1	30.59 (13.93–45.56)	2.57–180.04	2.07 (0.84–5.09)	0.115	0.98 (0.14–6.62)	0.981
	Day 7	14.16 (8.0–28.18)	0.69–102.91	1.0 (0.40–2.50)	1.0		

Me, median; IQR, interquartile range; Min, minimum; Max, maximum; OR, odds ratio; IL-1β, Interleukin 1 beta; IL-2, Interleukin 2; IL-6, Interleukin 6; IL-10, Interleukin 10; IL-12p70, Interleukin 12p70; IL-17A, Interleukin 17A; IFN-*γ*, Interferon gamma; GM-CSF, Recombinant human Granulocyte macrophage colony-stimulating factor; TNF α, tumor necrosis factor alpha; IL-18, Interleukin 18. Two models were made (day 1 and day 7). Multivariate analysis was performed in a logistic regression model with the cytokines after being dichotomized, taking as cut-off points the medians with a statistical significance of <0.25.

Bold values indicate statistical significance (*p* < 0.05).

**FIGURE 2:**
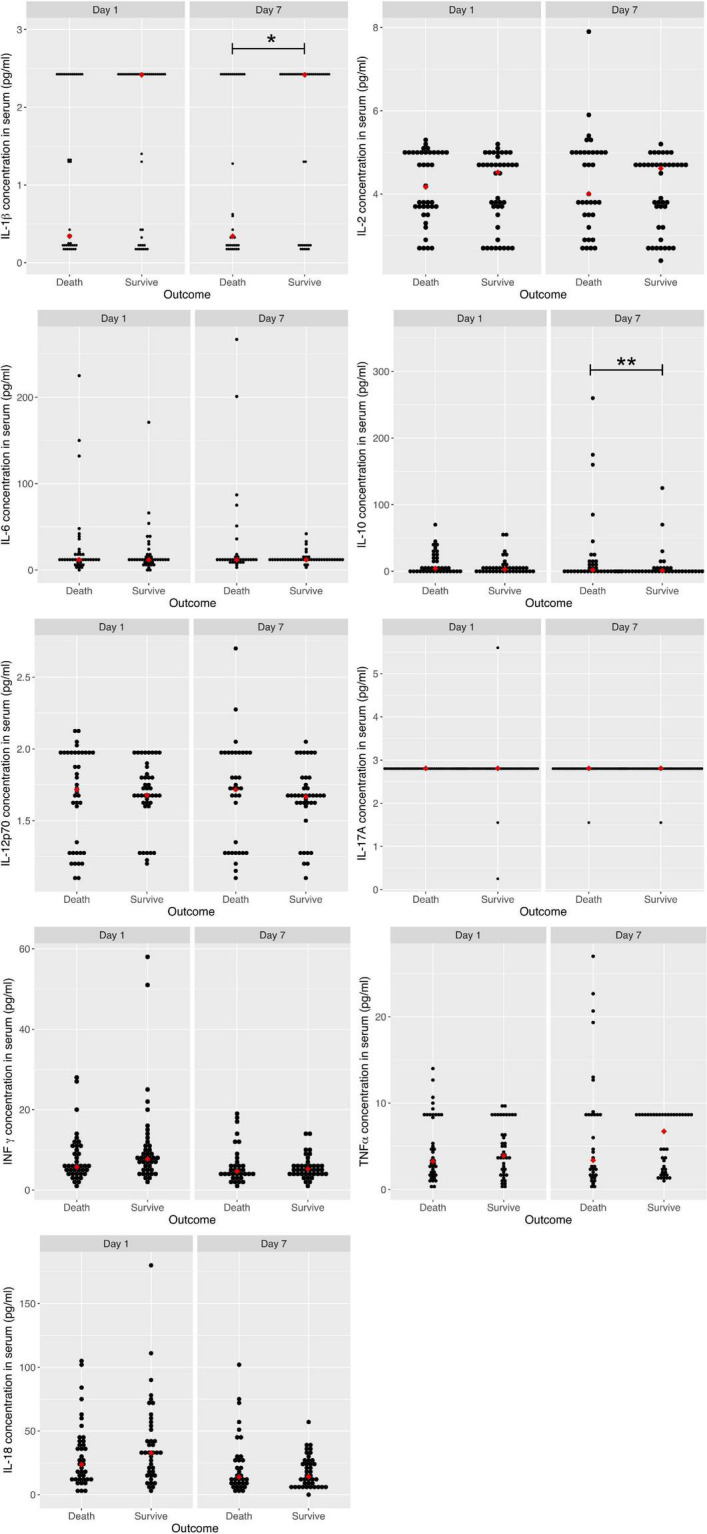
Serum cytokine levels on days 1 and 7 between COVID-19 patients who died and survived. IL-1β, Interleukin 1 beta; IL-2, Interleukin 2; IL-6, Interleukin 6; IL-10, Interleukin 10; IL-12p70, Interleukin 12p70; IL-17A, Interleukin 17A; IFN-*γ*, Interferon gamma; TNF α, tumor necrosis factor alpha; IL-18, Interleukin 18. *p-value < 0.05 for IL 1 β in serum cytokine concentrations on day 7 between who died and survived; **p-value < 0.01 for IL-10 in serum cytokine concentrations on day 7 between who died and survived.

When comparing the cytokine levels quantitatively, there were no differences between live and dead patients on day 1 (Figure 2). However, when dichotomizing the cytokines according to their median values, we found that IL-1β was associated with mortality, with an OR of 3.1 (1.24<7.71, *p* = 0.015). When performing a multivariate analysis with the cytokines after being dichotomized, the IL-1β cytokine continued with a statistical association, with an OR of 2.79 (1.08<7.22, *p* = 0.035), when the median levels were lower than 1.365 pg/ml (Table 2).

To determine the predictive value of mortality after the first week of hospitalization, we compared quantitative cytokine levels on day 7 of the patients who survived. Only IL-1β and IL-10 were associated with hospital mortality. The level of IL-1β was lower (0.34 vs. 2.41 pg/ml, *p* = 0.042) and that of IL-10 higher in patients who died (2.08 vs. 1.05 pg/ml, *p* = 0.009; Figure 2). When dichotomizing the cytokines according to their median values, we found that IL-1β was associated with mortality, with an OR of 3.14 (1.22<8.08, *p* = 0.017), and IL-12p70, with an OR of 0.36 (0.14<0.93, *p* = 0.034). When performing a multivariate analysis with the cytokines after being dichotomized, IL-12p70 continued with a statistical association, with an OR of 0.23 (0.07<0.73, *p* = 0.012), when median levels were higher than 1.666 pg/ml (Table 2).

Taking the change in the mean difference between the serum levels of each cytokine between days 1 and 7, we found decreases in both IFN-*γ* (3.939 pg/ml, *p* < 0.039) and IL-18 (16.312 pg/ml, *p* < 0.014), in patients who died (Table 3).

**TABLE 3:** Change in the mean difference between the serum levels cytokines between day 1 and day 7 with mortality.

Cytokines	Mean difference (pg/ml; SD) death	Mean difference (pg/ml; SD) survive	Mean difference (pg/ml) 95% CI	*p*
IL-1β	0.005 (0.442)	–0.041 (0.243)	0.046 (–0.116; 0.207)	0.574
IL-2	–0.134 (0.780)	0.023 (0.241)	–0.157 (–0.16; 0.101)	0.229
IL-6	–1.690 (52.465)	5.852 (28.609)	–7.542 (–26.819; 11.736)	0.438
IL-10	–15.054 (58.693)	–0.180 (18.354)	–14.874 (–36.410; 6.661)	0.170
IL-12p70	–0.021 (0.143)	0.029 (0.072)	–0.050 (–0.101; 0.002)	0.058
IL-17A	0.036 (0.211)	0.006 (0.667)	0.030 (–0.205; 0.265)	0.800
IFN-*γ*	1.650 (4.233)	5.589 (10.842)	–3.939 (–7.674; –0.204)	**0.039**
TNF α	–1.513 (6.647)	–1.019 (3.718)	–0.493 (–3.069; 2.083)	0.702
IL-18	7.259 (21.361)	23.571 (31.990)	–16.312 (–29.208; –3.416)	**0.014**

CI, confidence interval; SD, standard deviation; IL-1β, Interleukin 1 beta; IL-2, Interleukin 2; IL-6, Interleukin 6; IL-10, Interleukin 10; IL-12p70, Interleukin 12p70; IL-17A, Interleukin 17A; IFN-*γ*, Interferon gamma; TNF α, tumor necrosis factor alpha; IL-18, Interleukin 18.

Bold values indicate statistical significance (*p* < 0.05).

### IL-1β/IL-10 and TNFα/IL-10 ratios

**FIGURE 3:**
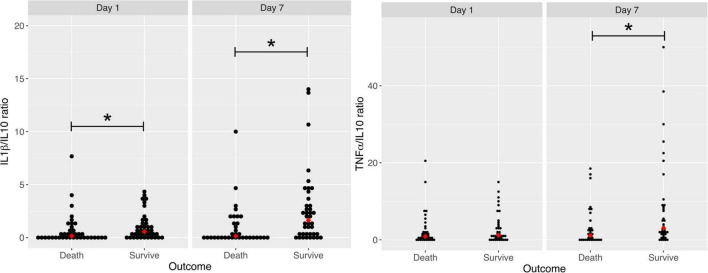
Comparison of IL-1β/IL-10 ratio and TNFα/IL-10 ratio concentrations on days 1 and 7 between COVID-19 patients who died and survived. TNF α, tumor necrosis factor alpha; IL-10, Interleukin 10; IL-1β, Interleukin 1 beta. For IL-1β/IL-10 ratio: *P value = 0.048 **P value = 0.002. TNFα/IL-10 ratio: *P value = 0.008.

A lower IL-1β/IL-10 ratio was associated with mortality on both days 1 and 7. The median on day 1 was 0.15 vs. 0.56 pg/ml (*p* = 0.048) and on day 7 0.14 vs. 1.63 (*p* = 0.002). A low TNFα/IL-10 ratio was associated with mortality only on day 7 (median 1.13 vs. 3.12 pg/ml, *p* = 0.008; Figure 3). The IL-1β/IL-10 ratio on day 7, with a cut-off below 2 [OR = 2.09 (1.05–4.12)], remained significantly associated with decreased survival after adjustments; however, the IL-1β/IL-10 ratio on day 1 lost its statistical significance. The TNFα/IL-10 ratio was not associated with mortality, even when the cut-off was below 4 [OR = 1.76 (0.93–3.31)].

### Cytokine concentration correlations

We also determined the correlation of serum cytokine levels with APACHE and SOFA severity scores, with laboratory values taken on the first day of ICU admission, and with the probability of developing pulmonary thromboembolism during the ICU stay, with the probability of still being intubated on day 7, and with pulmonary coinfection, defined by positive result of the Biofire^®^ FilmArray^®^ Pneumonia Panel, or of the microbiological cultures of respiratory samples from patients.

Regarding the correlation with the severity scores, only IL-2, taken on the first day of ICU admission, correlated positively and weakly with SOFA (Table 4).

**TABLE 4:** Correlation matrix between cytokines with severity scores and laboratories at admission.

		L-1β	IL2	IL6	IL10	IL12p70	IL17A	IFN-*γ*	TNF α	IL18
Neutrophil and lymphocyte ratio	Spearman	0.257	–0.164	0.055	0.049	–0.227	–0.1	0.193	0.118	0.217
	*P* value	**0.021**	0.145	0.63	0.667	**0.042**	0.38	0.086	0.299	0.054
C-reactive protein (mg/dl)	Spearman	–0.001	–0.100	–0.072	–0.019	–0.045	–0.104	0.113	–0.184	0.110
	*P* value	0.990	0.380	0.528	0.871	0.694	0.362	0.321	0.105	0.335
Procalcitonin (ng/ml)	Spearman	0.148	–0.142	0.017	0.056	0.137	0.113	0.213	–0.093	0.221
	*P* value	0.231	0.251	0.890	0.655	0.269	0.361	0.083	0.454	0.073
Creatinine (mg/dl)	Spearman	–0.071	0.013	–0.086	0.094	0.089	0.092	–0.095	–0.140	–0.107
	*P* value	0.531	0.906	0.448	0.409	0.430	0.416	0.404	0.216	0.343
Troponin (ng/ml)	Spearman	–0.056	0.280	0.096	0.023	0.382	–0.114	0.220	–0.144	0.211
	*P* value	0.655	**0.024**	0.447	0.854	**0.002**	0.366	0.078	0.251	0.091
Ferritin (ng/dl)	Spearman	0.141	0.031	0.035	–0.091	0.082	0.025	–0.106	0.083	–0.188
	*P* value	0.233	0.797	0.771	0.443	0.488	0.834	0.372	0.487	0.111
D-dimer (ng/ml)	Spearman	0.123	–0.075	0.110	–0.078	–0.010	0.113	–0.014	0.122	0.015
	*P* value	0.283	0.516	0.339	0.497	0.933	0.326	0.906	0.288	0.900
APACHE II	Spearman	–0.042	0.120	–0.051	0.190	–0.053	0.177	–0.108	–0.140	–0.056
	*P* value	0.714	0.289	0.652	0.092	0.637	0.117	0.342	0.214	0.622
SOFA	Spearman	–0.134	0.361	0.029	0.031	0.077	0.071	–0.105	0.160	–0.108
	*P* value	0.254	**0.002**	0.804	0.795	0.512	0.548	0.375	0.174	0.362

IL-1β, Interleukin 1 beta; IL-2, Interleukin 2; IL-6, Interleukin 6; IL-10, Interleukin 10; IL-12p70, Interleukin 12p70; IL-17A, Interleukin 17A; IFN-*γ*, Interferon gamma; TNF α, tumor necrosis factor alpha; IL-18, Interleukin 18; APACHE II, Chronic Health disease Classification System II; SOFA, sequential organ failure assessment.

Bold values indicate statistical significance (*p* < 0.05).

Of the laboratory tests taken in the first 24 h of ICU admission, the only test that correlated with any cytokine measured on day 1 was troponin, which correlated with IL-2 and IL-12p70, both weakly and positively. Regarding inflammatory response markers, only IL-1β positively (but weakly) and IL-12p70 negatively (but weakly) correlated with the neutrophil and lymphocyte ratio. No cytokine correlated with C-reactive protein or procalcitonin.

With respect to medical complications during the stay, only IL-2, taken on the first day of ICU admission, correlated with the probability of developing pulmonary thromboembolism during the ICU stay. Neither cytokine correlated with the probability of still being intubated on day 7. Moreover, neither cytokine correlated with pulmonary coinfection (Table 5).

**TABLE 5:** Cytokine concentration and correlation with pulmonary coinfection, mechanical ventilation on day 7 and pulmonary thromboembolism.

Cytokines (Day 1)	Pulmonary thromboembolism			Pulmonary coinfection			Mechanical ventilation day 7		
	Yes Me (P25–P75)	No Me (P25–P75)	*P*-value	Yes Me (P25–P75)	No Me (P25–P75)	*P*-value	Yes Me (P25–P75)	No Me (P25–P75)	*P*-value
L-1β	2.41 (0.22–2.4)	1.32 (0.22–2.41)	0.46	0.33 (0.22–2.41)	2.41 (0.22–2.41)	0.25	1.4 (0.22–2.41)	1.42 (0.16–2.41)	0.7
IL-2	3.12 (2.7–3.59)	4.7 (3.67–5)	**0.001**	3.189 (3.35–5)	4.61 (3.66–5)	0.76	4.5 (3.5–5)	4.27 (3.65–5)	0.94
IL-6	10.6 (4.2–26.6)	11.76 (10.57–18.21)	0.58	11.76 (7.97–21.8)	11.76 (11.51–16.36)	0.63	11.76 (9.3–16.66)	12.68 (11.76–23.66)	0.06
IL-10	4.6 (1.69–26.42)	3 (1.38–8.76)	0.57	3.37 (1.8–10.99)	3 (1.17–8.92)	0.64	2.85 (1.35–16)	3.45 (1.59–5.51)	0.98
IL-12p70	1.67 (1.26–1.79)	1.71 (1.58–1.98)	0.47	1.77 (1.42–1.98)	1.67 (1.58–1.94)	0.58	1.67 (1.58–1.98)	1.79 (1.66–1.98)	0.5
IL-17A	2.8 (2.8–2.8)	2.8 (2.8–2.8)	0.84	2.8 (2.8–2.8)	2.8 (2.8–2.8)	0.7	2.8 (2.8–2.8)	2.8 (2.8–2.8)	0.82
IFN-*γ*	7.2 (3.8–10.43)	6.66 (4.67–10.7)	0.78	7.13 (5.39–9.65)	6.66 (4.48–11.4)	0.84	6.55 (4.62–10.05)	11.06 (4.48–21.58)	0.15
TNF-α	3.513 (2.21–4.8)	3.98 (1.92–8.64)	0.7	3.38 (1.41–5.1)	4.23 (2–8.64)	0.13	3.76 (2.06–8.64)	5.19 (1.22–8.64)	0.67
IL-18	29.132 (20.29–45.22)	31.71 (13.93–45.569	0.72	29.13 (13.89–45.97)	31.7 (13.93–45.56	0.68	27.66 (13.93–41.49)	53.81 (15.49–79.2)	0.16

IL-1β, Interleukin 1 beta; IL-2, Interleukin 2; IL-6, Interleukin 6; IL-10, Interleukin 10; IL-12p70, Interleukin 12p70; IL –17A, Interleukin 17A; IFN-*γ*, Interferon gamma; TNF α, tumor necrosis factor alpha; IL-18; Interleukin 18; Me: median; P25–P75: 25th and 75th percentiles.

Bold values indicate statistical significance (*p* < 0.05).

## Discussion

In our study, when comparing cytokine levels in their quantitative form, there were no differences between live and dead patients on day 1. However, in the multivariate analysis we found that IL-1β was the only cytokine associated with mortality in patients who had median levels lower than 1.365 pg/ml compared to those who had higher values.

The SARS-CoV-2 has been considered to trigger an increased activation of macrophages and natural killer (NK) cells with a fulminant release of cytokines known as “CS” (17). The over-production of IL-1β acts on its receptor in tissue macrophages for the generation of more cytokines, which can lead to a vicious cycle resulting in multiple organ dysfunction in the patient (18). Treatment with anakinra, a recombinant human IL-1Ra, which limits the binding of IL-1β with its receptor, has been studied to prevent respiratory failure in patients with COVID-19 (19). However, according to our results, patients with median levels >1,365 pg/ml have a better outcome, and therefore treatment with anakinra would not be useful. One possible explanation is that the peak of IL-1β is early, before severe symptoms of the disease appear, and thus in critically ill patients requiring IMV, blood concentrations of IL-1β are at low levels and are associated with mortality (20).

The soluble urokinase plasminogen activator receptor (suPAR) is increased earlier than IL-1β, and plasma suPAR ≥ 6 ng/ml have been used in the SAVE-MORE study to guide the use of anakinra in hospitalized patients without respiratory failure defined by the use of high-flow oxygen (HFO), non-invasive ventilation (NIV) or IMV (21). suPAR levels were not measured in our study. It would be interesting to explore its relationship with IL-1β and its association with mortality.

A recent meta-analysis showed that several biomarkers including 10 cytokines are associated with severity and mortality in COVID-19 (22). Herr mentioned that a classical “CS” as seen in other conditions was not observed (23). The results of our study are in keeping with this statement. We found no association between serum IL-6 levels and mortality, as other studies have reported (24, 25).

There were no differences between live and dead patients in their conditions of immunosuppression at admission (rheumatologic disease, neoplasm, HIV, cirrhosis and prior steroid use), or at the time of onset of symptoms. To determine which phase of the inflammatory response predominated in the patients, we analyzed the IL-1β/IL-10 and the TNFα/IL-10 ratios as biomarkers of the balance between key pro- to anti-inflammatory levels. The lowest IL-1β/IL-10 ratio was associated with mortality on days 1 and 7, however, when analyzing a value or cut-off point associated with mortality, only the IL-1β/IL-10 ratio on day 7 remained significantly associated with decreased survival. A TNFα/IL-10 lower ratio was associated with mortality only on day 7. Moreover, we did not find a cut-off point that was associated with mortality. These results suggest that anti-inflammatory activity was predominant.

One possible explanation is that the anti-inflammatory activity of IL-10 has greater value. We found that the IL-10 median levels were higher on day 7 of ICU stay in patients who died. A meta-analysis showed that higher levels of IL-10 are associated with severity and mortality in patients with COVID-19 (26). Dorgham et al. detected elevated levels of IL-10 in patients with IMV and requiring extracorporeal membrane oxygenation (ECMO) (27). Lu et al. proposed that a sudden and strong elevation of IL-10 could play a role in lung injury in COVID-19 patients (28). Balzanelli et al. proposed an alternative “Trojan Horse” hypothesis where people who suddenly died would have shown a viral/bacterial/fungi coinfection with possible low expression of IL-10 (29).

Another possible conclusion is that the inflammatory activity provided by IL-1β and TNFα of patients who died is already decreased. Although median levels of IL-1β lower than 1.365 pg/ml were associated with mortality, we did not find similar results with TNF. Anderberg et al. identified that the TNFα/IL-10 ratio was correlated with multiple organ failure and mortality (10). A study by Jia et al. found that among 107 patients, those who were in the ICU with COVID-19, had higher values of TNFα, which was an independent risk factor for death (30). The interaction of the SARS-CoV-2 with angiotensin-converting enzyme 2 (ACE2) in alveolar epithelial cells is made possible by TNF-α, so anti-TNFα therapy could have a therapeutic role (31, 32).

The macrophages and dendritic cells produce IL-12 to activate NK cells and induce the secretion of IFN-γ as a defense mechanism against SARS-CoV-2 infection. This occurs in the early stages of the disease to limit the spread of the virus (33). In a study comprising 95 patients who had less than 10 days of COVID-19 symptoms, lower IL-12 levels were found in patients with severe disease (34). Other reports link IL-12 with severity. For example, Moll-Bernardes et al. in 167 hospitalized hypertensive patients with COVID-19 discovered that IL-12p70 were strongly associated with progression of symptoms to more severe forms of the disease (35). Studies on the association of IL-12 (p70) with mortality in COVID-19 are more limited. The multivariate analysis found that IL-12p70 was the only cytokine associated with mortality on day 7 of ICU stay when median levels were >1.666 pg/ml, i.e., those patients with levels <1.666 pg/ml have a 23% greater probability of survive than those with higher values. All patients at day 7 had received steroids for the management of COVID-19, and no patients were treated with anakinra or tocilizumab.

The main difference between days 1 and 7 was a decrease in IFN-γ and IL-18 levels in patients who had died. Trouillet-Assant et al. observed that the elevation of IFN-α 2 occurred on the first week of symptom onset, and then tapered off in 26 critically ill patients with COVID-19. Patients who did not present high IFN-α 2 peaks required IMV and a longer ICU stay (36). With a median of 9 days of symptom onset from day 1 of stay, we would expect lower levels of IFN on day 7. Bülow Anderberg et al. found that blood concentrations of IFN γ were elevated at hospital admission, appearing 11 days after initial symptoms appeared in patients with respiratory failure (10). Hence, IFN γ levels may reflect the later stages of the disease. Tang et al. mentioned that IFN- γ had no role in the survival analyses of 71 patients, and its level was lower in critically severe patients compared to those with mild symptoms (37).

Macrophages produce IL-18 to stimulate the production of IFN-γ for optimal viral host defense (38). IL-18 release induces ferritin, explaining the hyperferritinemia. Satış et al. reported that IL-18 serum concentrations above the cut-off value of 576 pg/ml on admission were associated with an 11.7-fold increased risk of ICU admission, in 58 COVID-19 patients (39). Dorgham et al. detected decreased levels of IL-18 in ECMO patients who died (27).

Neutrophil/lymphocyte ratios have been associated with mortality in COVID-19 (40). We found that only IL-1β and IL-12p70 correlated with the neutrophil and lymphocyte ratio. No cytokine correlated with C-reactive protein or procalcitonin. Moreover, that troponin correlates with IL-2 and IL-12p70. Song et al. noticed that TNFα, IL-8 and IL-6, were higher in 64 critically ill patients with cardiac dysfunction (41).

The strengths of our study are listed as follows: (1) This is a multicenter Latin American study; (2) Our investigation comprised critically ill patients on mechanical ventilation; (3) The serum samples were taken early in the course of their hospitalization with follow-up samples taken on day 7, covering the dynamic changes of the disease; (4) We measured different correlations of cytokine concentration with laboratory tests, pulmonary thromboembolism and pulmonary coinfection.

The limitations of this study include: (1) We did not measure viral titers and therefore, do not know whether they correlate with cytokines; Guo et al. described that the virus titers were correlated with the cytokines (42); (2) The sample size could be considered small; (3) We do not know the relationship of serum cytokines with the immune and inflammatory response at the pulmonary level; (4) We did not measure macrophages, helper/inducible T lymphocyte (CD3+ CD4+ %) or inhibitory/cytotoxic T lymphocyte (CD3+ CD8+ %) levels to analyze its correlation with cytokines.

In conclusion, on day 1 of ICU stay, IL-1β was the only cytokine that has a statistical association, with an increase in mortality in those patients who had median levels <1.365 pg/ml. On day 7 of ICU stay, IL-12p70 was associated with mortality when median levels were >1.666 pg/ml and the IL-1β/IL-10 ratio cut-off was <2. A classical “CS” was not observed in this study. The inflammatory activity of patients who died was already decreased. Anakinra or tocilizumab therapy might not be useful in our patients.

## Data availability statement

The original contributions presented in this study are included in the article/supplementary material, further inquiries can be directed to the corresponding author.

## Ethics statement

The studies involving human participants were reviewed and approved by Universidad Pontificia Bolivariana Ethics Committee. The patients/participants provided their written informed consent to participate in this study.

## Author contributions

All authors listed have made a substantial, direct, and intellectual contribution to the work, and approved it for publication.
